# Using community volunteers to promote exclusive breastfeeding in Sokoto State, Nigeria

**DOI:** 10.4314/pamj.v10i0.72215

**Published:** 2011-09-23

**Authors:** Asma Misbah Qureshi, Oche Mansur Oche, Umar Abubakar Sadiq, Sabitu Kabiru

**Affiliations:** 1 Consultant, University Research Co. LLC, Bethesda MD, USA; 2 Department of Community Health, Usmanu Danfodiyo University, Sokoto, Nigeria; 3 Ahmadu Bello University, Zaria, Nigeria

**Keywords:** Exclusive Breast Feeding, community interventions, health promotion, Nigeria

## Abstract

**Background:**

Exclusive Breastfeeding (EBF) refers to the practice of feeding breast milk only, (including expressed breast milk) to infants; and excluding water, other liquids, breast milk substitutes, and solid foods. Inadequately breastfed infants are likely to be undernourished and have childhood infections. EBF knowledge and infant feeding practices have not been studied sufficiently in Sokoto State, Nigeria. We describe the results of a randomized community trial to promote Exclusive Breastfeeding (EBF) in two local government areas Kware and Bodinga selected as intervention and control groups respectively.

**Methods:**

During advocacy meetings with community leaders, a Committee was formed. Members of the Committee were consulted for informed consent and selection of ten female volunteers who would educate mothers about breastfeeding during home visits. Participants comprised mothers of infants who were breastfeeding at the time of the study. A total of 179 mothers were recruited through systematic random sampling from each community. Volunteers conducted in-person interviews using a structured questionnaire and counseled mothers in the intervention group only.

**Results:**

At baseline, intervention and control groups differed significantly regarding maternal occupation (P=0.07), and age of the index child (P=0.07). 42% of infants in the intervention group were up to 6 months old and about 30% of them were exclusively breastfed. Intention to EBF was significantly associated with maternal age (P=0.01), education (P=0.00) and women who were exclusively breastfeeding (P=0.00). After counseling, all infants up to 6 months of age were exclusively breastfed. The proportion of mothers with intention to EBF increased significantly with maternal age (P=0.00), occupation (P=0.00) and women who were exclusively breastfeeding (P=0.01). Post-intervention surveys showed that source of information and late initiation of breastfeeding was not significantly associated with intention to EBF. Mothers who reported practicing EBF for 6 months, were older (P=0.00) multi-parous (P=0.05) and more educated (P=0.00) compared to those who did not practice EBF. Among them, significantly increased proportion of women agreed that EBF should be continued during the night (P=0.03), infant should be fed on demand (P=0.05), sick child could be given medication (P=0.02), EBF offered protection against childhood diarrhea (P=0.01), and helped mothers with birth spacing (P=0.00).

**Conclusion:**

This study shows that there is a need for reaching women with reliable information about infant nutrition in Sokoto State. The results show decreased EBF practice among working mothers, young women, mothers with poor education and fewer than five children. Counseling is a useful strategy for promoting the duration of EBF for six months and for developing support systems for nursing mothers. Working mothers may need additional resources in this setting to enable them to practice EBF.

## Background

Under-five mortality rate (U5MR) is a sensitive indicator of national development and is being used to monitor MDG 4: to reduce by two-thirds, between 1990 and 2015, the mortality rate of children under five. Globally, under-nutrition is responsible for more than one-third of all U5MR [1] frequently resulting from food deficiency and poor quality, limited access to clean water, sanitation and health services, and suboptimal feeding practices. Approximately three quarters of U5MR is concentrated in ten countries of Africa and Asia including India, Bangladesh, Pakistan, China, Nigeria, Ethiopia, Indonesia, Democratic Republic of Congo, Philippines, and Vietnam [2]. Although progress toward achieving Millennium Development Goals (MDG) 4 has been insufficient in many countries, it is to be noted that in West and Central Africa, there has been no progress toward achieving this goal [2.]

Malnutrition remains a major contributing cause of infant morbidity [2, 3]. In sub-Saharan Africa, deaths due to under-nutrition account for approximately 35% of all U5MR [3] and are associated with stunting, severe wasting, low birth weight and micronutrient deficiencies. Inadequate breastfeeding practices further compound the problem. A central factor related to this issue is maternal health and nutritional status [4]. Given the close relationship between maternal mortality and neonatal mortality, The Saving Newborn Lives Initiative focuses on an integrated postnatal care strategy to improve the chances of survival for both mothers and their babies [5]. Studies have shown that up to 70% of newborn deaths can be averted by ensuring clean delivery, care of low birth weight babies by providing warmth, early initiation of exclusive breastfeeding, hygienic eye and cord care, and early recognition and treatment of illness [5].

Even in the poorest countries, Exclusive Breastfeeding (EBF) is well-known as an affordable and feasible intervention that improves newborn survival. It fulfills the nutritional requirements of infants and protects them from childhood infections including diarrhea, and pneumonia [6, 7]. EBF associated with Lactational Amenorrhea Method (LAM) is an important choice for postpartum family planning [8]. Conversely, mothers who do not breastfeed are more likely to develop post-partum depression, obesity, type 2 Diabetes Mellitus, breast cancer and hypertension [9]. Promotion and sustainability of exclusive breastfeeding practices and LAM in the community is a crucial step in improving the nutritional status of children under the age of five years [10]. Studies show that community health workers can be successfully trained to perform tasks related to child survival [11]. An example of a successful program is Pakistan's Lady Health Workers Program which began in 1994 and provided maternal and child health services to underserved communities in rural areas and urban slums. In addition to facilitating women's health committees, maintaining linkages with traditional birth attendants, treating minor illnesses, supplying condoms and oral contraceptives, Lady Health Workers provided counselling related to breastfeeding, complementary feeding and immunizations [11].

**Figure 1:**
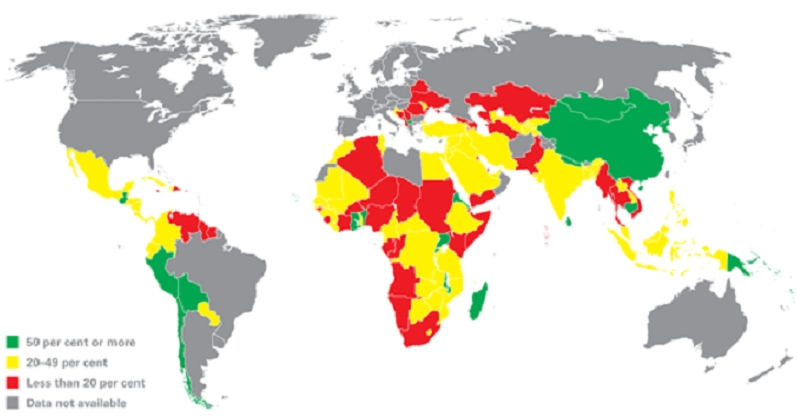
Worldwide distribution (%) of children less than six months old exclusively breastfed (2000-2006)

Current recommendations on optimal breastfeeding include initiation of breastfeeding within the first hour after birth; exclusive breastfeeding for the first six months and continued breastfeeding for up to two years or more with safe and appropriate complementary feeding beginning in the sixth month [12] (Figure 1). Despite methodological issues regarding research on the optimal duration of EBF, a review of clinical trials and observational studies from developing countries including Nigeria strongly suggests that EBF for up to six months offers significant health benefits to both mothers and babies [12]. However, EBF without iron supplementation beginning at 3 to 4 months of age may lead to anemia, which may require oral vitamin drops [12]. The Innocenti Declaration on the Protection, Promotion and Support of Breastfeeding affirms the role of EBF in fulfilling the basic human rights of the child to attain the highest standards of health [13]. Given that prevalence of EBF varies across regions and that breastfeeding is central to alleviating hunger, EBF practice links MDG 4 with MDG 1: halve, between 1990 and 2015, the proportion of people who suffer from hunger [14]. Studies confirm that enhancing women's empowerment, education and decision-making abilities are strongly related to newborn survival [15].

At the health system level, one of the targets of The Innocenti Declaration is to enhance health worker competencies and appropriate maternity services to promote optimal breastfeeding: the Baby-Friendly Hospital Initiative and the Baby-Friendly Community Initiative (Table 1) [16]. In Nigeria, there are currently 1147 Baby Friendly facilities, the largest number in West and Central Africa region [17]. Women who deliver in these health facilities are less likely to practice traditional prelacteal feeding and more likely to initiate exclusive breastfeeding within the first hour after birth, compared to women who deliver at home [18] (Table 2). In order to expand services and reach poor women, community volunteers are being trained to promote breastfeeding and to engage communities in reducing infant morbidity and mortality.

**Table 1:** UNICEF UK: Baby Friendly Community Initiative Poster

1. Have a written breastfeeding policy that is routinely communicated to all health-care staff.
2. Train all staff involved in the care of mothers and babies in the skills necessary to implement the policy.
3. Inform all pregnant women about the benefits and management of breastfeeding.
4. Support mothers to initiate and maintain breastfeeding.
5. Encourage exclusive and continued breastfeeding, with appropriately-timed introduction of complementary foods.
6. Provide a welcoming atmosphere for breastfeeding families.
7. Promote co-operation between healthcare staff, breastfeeding support groups and the local community.

Source: http://www.babyfriendly.org.uk/pdfs/seven_point_plan_poster.pdf

**Table 2:** Context of Malnutrition in Nigeria 2003-2008

**Breastfeeding indicators**	
Early initiation of breastfeeding	32%
Exclusive Breast Feeding up to 6 months	13%
Continued breastfeeding with complementary foods up to 9 months	75%
	
**Nutrition indicators**	
Preventing Vit A deficiency through supplementation (6-59 months)	74% coverage rate
Preventing Iodine deficiency through use of iodized salt	97% of households
Prevalence of moderate to severe stunting	41%
Prevalence of moderate to severe wasting	14%
Prevalence of underweight	23%
Infants with Low birth weight	14%
	
**Other child health indicators**	
Estimated number of children (aged 0-14 years) living with HIV in 2007	220,000
U5MR in 2008	186
Average annual rate of reduction in U5MR (1990-2008)	1.2%
Reduction since 1990	19%

### Definitions

The following definitions are taken from WHO [19]:

**Exclusive Breastfeeding (EBF)** is defined as the practice of feeding breast milk only, including expressed breast milk, to infants and excluding water, other liquids, breast milk substitutes, and solid foods. Vitamin drops, minerals, oral rehydrating solution (ORS) and medicines may be given.

**Predominant Breastfeeding** is defined as the practice of feeding breast milk only, including expressed breast milk, to infants as the predominant source of nutrition. Certain liquids including water, water-based liquids, juices, ritual fluids, vitamin drops, minerals, oral rehydrating solution (ORS) and medicines may be given.

**Partial breastfeeding** is defined as the practice of feeding breast milk only, including expressed breast milk, to infants in addition to food or liquids including non-human milk and formula.

**“Any breastfeeding”** includes exclusive breastfeeding, predominant breastfeeding and partial breastfeeding.

**Prelacteal feeds** are defined as feedings that are given to the infant before they are put to the breast for the first time.

### Context

The following information is taken from DHS 2003 survey data [18]: Sokoto State is located in the North West region of Nigeria. 75% of women in the North West are uneducated and one-third of children are severely stunted. There is a positive relationship between antenatal care and educational level. Almost 89% of deliveries take place at home. Estimates show that 59% of women in Northern Nigeria compared to women in the South and almost half of teenage mothers compared to one-third of mothers over 20 years of age, do not receive ante-natal care. Exposure to media is low in Northern Nigeria, in rural areas and among older women while education and wealth are positively correlated with mass media exposure. Although radio is the most commonly used media source, approximately 27% of women in the North West have no exposure to newspaper, TV and radio. About 68% of women are traders and about 58% have no participation in decision making. Breastfeeding is universally accepted in Nigeria yet only one-third of babies are put to the breast within one hour of birth. In the North West, only half of infants are given breast milk within one hour of birth. Low maternal education and poverty are directly related to delayed initiation of breastfeeding and prelacteal feeding. 98% of infants in the North West are ever breastfed and among them, 79% of infants receive a prelacteal feed. Overall, median duration of any breastfeeding in Nigeria is 18.6 months but EBF lasts only half a month because of early introduction of water, breast milk substitutes and other liquids.

### Culture

Studies from Zambia [20], Nigeria [21], Ghana [22] and Ethiopia [23] show that early introduction of water and other liquids to infants less than six months of age is acceptable and prelacteal feeds are the norm in these countries. In northeastern Nigeria the rationale for this practice is that a newborn child is thirsty [24]. There is a widely held belief that breast milk alone is insufficient for infants and breastfeeding during pregnancy can harm a developing fetus [24]. Grandmothers and traditional birth attendants often force mothers to discard colostrum [24]. Although fathers and grandmothers consider breast milk as “good” nutrition for the infant, breastfeeding in public is generally opposed by fathers [24]. Home visits and radio are the best methods for reaching women who are usually confined to their homes after marriage [24].

Among rural women in Zambia, fear of dying is reported as a major barrier to Exclusive Breastfeeding and these women rely on older relatives for information about breastfeeding [20]. In Nigeria, women report that colostrum is dirty “like pus” and expressed milk is susceptible to witchcraft and poisoning [21].

EBF knowledge and infant feeding practices have not been studied sufficiently in Sokoto State, Nigeria. We describe the results of a randomized community trial to promote Exclusive Breastfeeding (EBF) in two local government areas Kware and Bodinga selected as intervention and control groups respectively. Both communities share similar socio-demographic variables. The total population of Kware is 134,084 while that of Bodinga is 174,302 [19]. In Kware, maternal and child health services are provided by a Primary Health Centre as well as a Comprehensive Health Centre operated by Usmanu Danfodiyo University Teaching Hospital, Sokoto.

## Methods

### Study Design

Participants included biological mothers who were breastfeeding at the time of study. In the initial phase, a series of advocacy visits to community and opinion leaders were conducted. During these meetings, a Committee was formed, and the strategy was discussed. Ten female volunteers were nominated with these minimum qualifications: primary school certificate, previous breastfeeding experience, residence in the community and willingness to teach mothers about breastfeeding. Volunteers were trained at a four-day workshop held in Kware comprising lectures, role plays and demonstrations using posters and flip charts. Each session lasted two and a half hour. Training content covered counseling skills, the basics of nutrition, exclusive breastfeeding and the survey instrument.

In the second phase of the study the survey instrument was pre-tested in a similar community in neighboring Kebbi state. Results showed that 19% of women in the sample were exclusively breastfeeding and 45% had adequate knowledge of EBF after counseling. This pilot study was followed by baseline surveys in Kware and Bodinga, where volunteers conducted in-person interviews and collected data on socio-demographic characteristics, attitudes, maternal knowledge and infant feeding patterns including exclusive breastfeeding. Post-intervention surveys were conducted six months after counseling using the same questionnaire.

Using a combination of simple and systematic sampling methods, a one in eight sample of 179 mother-child pairs were recruited from each community. In homes without breastfeeding mothers at the time of counting, the next house was chosen. Given the existence of polygamy in Sokoto, whenever a situation was encountered in which there were more than one mother child pairs in a single house, one pair was selected by simple random sampling using the balloting technique.

Data were analyzed and preliminary findings were discussed with the Committee. An action plan was drawn up regarding implementation of counseling activities, and Principal Investigators discussed with Committee members, the advantages of breastfeeding, the myths and difficulties related to breastfeeding practice, importance of the “ten steps to successful breastfeeding”, cultural beliefs about pre-lacteal feeding, essential foods that improve nutritional status of mothers, and significance of adequate rest and personal hygiene for nursing mothers. Additionally, community leaders were informed about the critical need for developing community support for breastfeeding. Follow-up visits were conducted by volunteers to investigate whether participants had further concerns regarding breastfeeding.

### Ethics

Informed consent was obtained from all participants. The study was approved by Usmanu Danfodio University Institutional Review Board.

### Measurement

Chi-square statistical test was used; and sample size was calculated using the two-sided test with a level of significance of 0.05; and approximately 90% power (β=0.1) for detecting the difference of 15% to 30% in knowledge of EBF by demographic characteristics [25].

Independent variables included socio-demographic characteristics of mothers, and knowledge of infant feeding practices.

Participants’ knowledge and practice of EBF were assessed using a modified questionnaire based on Community Level Nutrition Information System for Action (COLNISA) [26]. The questionnaire was translated from English to Hausa and back translated to English. Local scholars participated in translation of the survey questionnaire to Hausa. The modified instrument consisted of four questions about attitudes related to EBF and eight questions about benefits of breastfeeding. The questionnaire also collected demographic information including age, parity, education, religion, occupation, husband's occupation, age and sex of the index child. Yes or no responses were coded by interviewers. Each correct response was given a score of 1 and each incorrect response was given a score of 0. Correct responses were based on information provided during counseling. Scores were graded as percentage with a cut-off at 50% to represent adequate knowledge. Participants were stratified into two groups based on their level of knowledge. “Intention to EBF” was defined as knowledge score of >50%. Mothers with knowledge score of <50% were classified as “No intention to EBF”. Data was analyzed using EPI INFO 3.3.

## Results

### Characteristics of mothers and infants

Table 3 shows that the majority of participating mothers were multiparous. The average number of children was 5. Approximately 20% of mothers in either group were under the age of 20 years and about 50% were between 23–32 years old.

**Table 3:** Characteristics of mothers and infants in Kware (intervention) and Bodinga (control) communities (N= 179 in each group)

	KWARE	BODINGA
	
	No. (%)	No. (%)
	
Parity	5	5
**Maternal Age (years)**		
13-22	39 (22)	30 (17)
23-32	84 (47)	89 (50)
>33	56 (31)	60 (33)
	P=0.5	
		
**Age of index child (months)**		
0-6	75 (42)	66 (37)
7-12	50 (28)	46 (26)
13-18	26 (15)	33 (18)
19-24	13 (7)	16 (9)
≥ 24	15 (8)	18 (10)
	P=0.07	
		
**Occupation**		
Housewife	109 (61)	124 (69)
Civil servant	21 (12)	24 (13)
Trader	49 (27)	31 (17)
	P=0.07	
		
**Level of Education**		
Non-formal		
None	60 (34)	53 (30)
Arabic	57 (32)	49 (27)
Formal		
Primary	33 (18)	38 (21)
Secondary	20 (11)	26 (15)
Post-secondary	9 (5)	13 (7)
	P=0.6	
		
**Initiation of Breastfeeding**		
<30 minutes	94 (53)	88 (49)
>30min–6hours	34 (19)	29 (16)
>6hours–24hours	17 (9)	23 (13)
>24 hours	34 (19)	39 (22)
	P=0.6	
**Exclusively Breastfeeding**	55 (31)	40 (22)
	P=0.1	
		
**Stopped Breastfeeding**		
Infant age (months)		
0–5	1 (1)	0
6-10	2 (1)	6 (3)
11–15	5 (3)	17 (10)
Up to 24 months	45 (25)	(38)
>24 months	126 (70)	88 (49)
	P=0.00	

Although the majority of women in either community were housewives, approximately 40% of women in Kware (intervention group) and 30% of women in Bodinga (control group) were working as civil servants and traders. This finding was statistically significant (P=0.07). Educational attainment was low in both communities; 34% of women in Kware compared to 43% of women from Bodinga had completed formal education. In both communities, approximately half of the participants initiated breastfeeding within thirty minutes and approximately 20% initiated breastfeeding after 24 hours.

The difference in age of the index child between both communities was statistically significant (P=0.07). 42% of infants in Kware and 37% of infants in Bodinga were up to six months old; however, only 30% of infants in Kware and 20% of infants in Bodinga were exclusively breastfed. In either community about 30% of infants were up to 12 months old; 18% of infants in Bodinga were up to 18 months old versus 15% of infants in Kware; and about 20% of infants in Bodinga were more than 18 months old versus 15% in Kware. In this sample, mother-child pairs residing in Bodinga (control group) were older than mother-child pairs in Kware (intervention group). No discontinuation in breastfeeding was noted in the first four months except for one mother in Kware who stopped breastfeeding due to a new pregnancy. The discontinuation rate in the first 24 months was statistically significantly higher in the control group compared to the intervention group (Bodinga: 50%; Kware: 29.6%; P=0.00).

### Intention to exclusive breastfeeding

Table 4 shows the demographic characteristics of mothers with intention to EBF before and after counseling. Intention to EBF was defined as knowledge score of >50%. Pre-intervention assessment showed that the proportion of mothers with Intention to EBF increased significantly with maternal age (P=0.01), education (P=0.00) and women who were exclusively breastfeeding (P=0.00). After counseling, the proportion of mothers with intention to EBF increased significantly with maternal age (P=0.00), occupation (P=0.00) and women who were exclusively breastfeeding (P=0.01). The proportion of mothers with non-formal education who planned to breastfeed exclusively increased compared to those with formal education but the difference was not statistically significant.

**Table 4:** Characteristics of mothers in Kware (intervention group) with intention to practice exclusive breast feeding (EBF) (N=179)

	Pre-intervention		Post-intervention	
	
	Intention to EBF	No intention to EBF	Intention to EBF	No intention to EBF
	*No. (%)*	*No. (%)*	*No. (%)*	*No. (%)*

**Parity**				
<5	21 (12)	66 (37)	46 (25)	40 (23)
>5	33 (18)	59 (63)	61 (75)	32 (77)
Total	54 (30)	125 (70)	107 (60)	72 (40)
	P=0.1		P=0.1	
				
**Maternal age (Mean age=29.8 years)**				
<30 years	21 (12)	76 (42)	47 (26)	50 (28)
>30 years	33 (18)	49 (28)	60 (34)	22 (12)
Total	54 (30)	125 (70)	107 (60)	72 (40)
	P=0.01	P=0.00		
				
**Level of Education**				
Non-formal	13 (7)	104 (58)	79 (44)	38 (21)
Formal	34 (19)	28 (16)	41 (23)	21 (12)
Total	47 (26)	132 (74)	120 (67)	59 (33)
	P=0.00		P=0.4	
				
**Occupation**				
Housewife	33 (18)	76 (43)	51 (29)	58 (32)
Civil servant/trader	21 (12)	49 (27)	56 (31)	14 (8)
Total	54 (30)	125 (70)	107 (60)	72 (40)
	P= 0.1		P=0.00	
Exclusively Breastfeeding	29 (16)	26 (15)	53 (30)	2 (1)
Not Exclusively Breastfeeding	25 (14)	99 (55)	54 (30)	70 (39)
Total	54 (30)	125 (70)	107 (60)	72 (40)
	P=0.00	P=0.01		

Results from post-intervention surveys in Table 5 show that source of information and reasons for late initiation of breastfeeding were not significantly associated with intention to EBF. Notably, none of the mothers in the sample reported husbands as their source of information.

**Table 5:** Post-intervention survey: Selected responses about attitude toward lactation by intention to practice exclusive breast feeding (EBF) (N=179)

	Intention to EBF	No intention to EBF
	
	No (%)	No (%)

**Source of information**		
Hospital	20 (11)	12 (7)
Friend	19 (11)	17 (9)
Husband	0 (0)	0 (0)
Media	12 (7)	9 (5)
Grandmother	56 (31)	34 (19)
Total	107 (60)	72 (40)
	P=0.7	
		
**Late initiation of breastfeeding**		
Colostrum is dirty	44 (25)	27 (15)
Insufficient milk	20 (11)	23 (13)
Mother's illness	19 (10)	10 (6)
Child's illness	14 (8)	8 (4)
Other	10 (6)	4 (2)
Total	107 (60)	72 (40)
	P=0.3	

### Benefit of counselling

Table 6 shows demographic characteristics of mothers who reported breastfeeding up to six months after counseling. 75 infants (42%) in Kware were up to six months old and all were exclusively breastfed. The proportion of mothers who were practicing EBF increased significantly with parity (P= 0.05), maternal age (P=0.00), and education (P=0.00). In this setting, older mothers with more than five children who have completed formal education are more likely to practice EBF.

**Table 6:** Post-intervention survey: Characteristics of mothers in Kware (intervention group) practicing exclusive breast feeding (EBF) up to 6 months (N=179)

	Practicing EBF	Not practicing EBF
	
	No. (%)	No. (%)

**Parity**		
<5	22 (12)	49 (27)
>5	53 (30)	55 (31)
Total	75 (42)	104 (58)
	P=0.05	
		
**Maternal age (Mean age=29.8 years)**		
<30 years	36 (20)	75 (42)
>30 years	39 (22)	29 (16)
Total	75 (42)	104 (58)
	P=0.00	
		
**Level of Education**		
Non-formal	27 (15)	90 (50)
Formal	48 (27)	14 (8)
Total	75 (42)	104 (58)
	P=0.00	
		
**Occupation**		
Housewife	50 (28)	59 (33)
Civil servant/trader	25 (14)	45 (25)
Total	75 (42)	104 (58)
	P=0.25	

Table 7 shows selected responses about perceived benefits of EBF among these mothers. Significantly increased proportion of women agreed that EBF should be continued during the night (P=0.03), infant should be fed on demand (P=0.05), sick child could be given medication (P=0.02), EBF offered protection against childhood diarrhea (P=0.01), and helped mothers with birth spacing (P=0.00).

**Table 7:** Post-intervention survey: Selected responses about perceived benefits of lactation by exclusive breast feeding (EBF) status (N=179)

	Practicing EBF	Not practicing EBF
	
	No. (%)	No. (%)

**Protection against infection and malnutrition**		
Yes	41 (23)	59 (33)
No	34 (19)	45 (25)
Total	75 (42)	104 (58)
	P=0.9	
		
**Protection against diarrhea**		
Yes	50 (28)	48 (27)
No	25 (14)	56 (31)
Total	75 (42)	104 (58)
	P=0.01	
		
**Birth spacing**		
Yes	59 (33)	23 (13)
No	16 (9)	81 (45)
Total	75 (42)	104 (58)
	P=0.00	
		
**Feeding at night**		
Yes	49 (27)	50 (28)
No	26 (15)	54 (30)
Total	75 (42)	104 (58)
	P=0.03	
		
**Feeding on demand**		
Yes	50 (28)	53 (30)
No	25 (14)	51 (28)
Total	75 (42)	104 (58)
	P=0.05	
		
**Medication can be given**		
Yes	54 (30)	56 (31)
No	21 (12)	48 (27)
Total	75 (42)	104 (58)
	P=0.02	

## Discussion

This study is the first randomized trial to promote Exclusive Breast Feeding (EBF) in Sokoto State. The objective is to describe whether counseling by community health volunteers is beneficial in improving maternal knowledge of EBF and infant feeding practices. It is assumed that participants are residents of the North West region and the sample is representative of all women living in this area.

The socio-demographic characteristics of study participants are fairly typical of women in the North West region where poor education, high fertility, and low socio-economic status are prevalent [18]. The study sample comprised housewives, working women, multi-parous women, and few women with formal education. Teen marriages are not uncommon in this setting; approximately 20% of the mothers in either group were under the age of 20 years.

Data from the study show that approximately 20% of mothers initiated breastfeeding after 24 hours suggesting the underlying perception that colostrum is dirty. Although, most women in Kware and Bodinga continued to breastfeed beyond 24 months, less than one third of participants in either community practiced Exclusive Breastfeeding. This indicates a potential role for appropriate education so that mothers can successfully learn about Exclusive Breastfeeding. In both groups, mothers continued to breastfeed their infants for four months. Only one mother reported discontinuing breastfeeding due to a new pregnancy possibly due to the belief that breastfeeding in pregnancy is harmful to the developing fetus.

Approximately 60% of mothers in Kware and 70% of mothers in Bodinga were housewives suggesting that they are not equal partners in decision making. A study on Healthy Timing and Spacing of Pregnancy conducted in Kano State showed that women perceive themselves as isolated, without access to information and unable to express their opinions [27].

Additionally, mothers in this sample are more likely to be confined to their homes and to have delivered at home, further compounding the problem of receiving EBF information from a health worker [28]. Results of this study show that counseling via home visits, increased the duration of EBF for six months. This indicates the existence of information asymmetry and the need for reaching women with reliable information about infant nutrition. None of the participants reported husbands as their source of information suggesting that husbands are unable to communicate factual information about EBF. Mothers who were exclusively breastfeeding agreed that birth spacing and protection against childhood diarrhea were benefits of EBF. This is important in poor communities where the prevalence of diarrhea is high and there are inequities in access to family planning/reproductive health services.

Several studies from Nigeria have reported on EBF outcomes in various regions [29–39]. An analysis of DHS 2003 data by Agho et al [30] showed that the odds of EBF practice were lower in the North West and North East region compared to all regions of Nigeria.

Lessons learned from research and program implementation worldwide show that counseling by community health volunteers is critical in improving EBF outcomes [40]. This finding underscores the importance of breastfeeding support groups in the community especially for young mothers, and those who are under stress and more likely to believe that breast milk is insufficient [40]. Data analysis shows decreased EBF practice among working mothers, young women, mothers with poor education and fewer than five children (occupation: P=0.25; age: P=0.00; education: P=0.00; parity: P=0.05). Involving the community leaders helps to shift cultural norms related to EBF practices in that community and may potentially influence women's empowerment [41–44].

Data were collected and analyzed in 2006. The timing of the study and characteristics of mother-child pairs are important limitations of this study. In Bodinga (control group), mother-child pairs were older than in Kware (intervention group). Data collection relied on interviews by community health volunteers. Their presence may have influenced responses or participants may have experienced recall bias. More rigorous data analysis would have enhanced the study.

## Conclusion

This study shows that there is a need for reaching women with reliable information about infant nutrition in Sokoto State. The results show decreased EBF practice among working mothers, young women, mothers with poor education and fewer than five children. Counseling is a useful strategy for promoting the duration of EBF for six months and for developing support systems for nursing mothers. Working mothers may need additional resources in this setting to enable them to practice EBF.
